# Cost of Hospitalization Associated with Inpatient Goals-of-Care Program Implementation at a Comprehensive Cancer Center: A Propensity Score Analysis

**DOI:** 10.3390/cancers16071316

**Published:** 2024-03-28

**Authors:** David Hui, Yu-Ting Huang, Clark Andersen, Brian Cassel, Nico Nortje, Marina George, Eduardo Bruera

**Affiliations:** 1 Department of Palliative, Rehabilitation and Integrative Medicine, The University of Texas MD Anderson Cancer Center, Houston, TX 77030, USA; ebruera@mdanderson.org; 2 Cost Management and Decision Support, The University of Texas MD Anderson Cancer Center, Houston, TX 77030, USA; yuting@mdanderson.org; 3 Department of Biostatistics, The University of Texas MD Anderson Cancer Center, Houston, TX 77030, USA; crandersen@mdanderson.org; 4 Hematology/Oncology & Palliative Care, Virginia Commonwealth University, Richmond, VA 22043, USA; brian.cassel@vcuhealth.org; 5 Section of Integrated Ethics, Department of Critical Care Medicine, The University of Texas MD Anderson Cancer Center, Houston, TX 77030, USA; nnortje@mdanderson.org; 6 Department of Dietetics and Nutrition, University of the Western Cape, Bellville 7535, South Africa; 7 Department of Hospital Medicine, Division of Internal Medicine, The University of Texas MD Anderson Cancer Center, Houston, TX 77030, USA; mgeorge1@mdanderson.org

**Keywords:** communication, healthcare costs, intensive care units, neoplasms, palliative care, patient care planning, quality of healthcare

## Abstract

**Simple Summary:**

Goals-of-care discussions, by promoting mutual understanding between patients and clinicians, supporting decision making and facilitating care planning, have been found to reduce acute care use and cost of care downstream. However, it is not known if goals-of-care discussions have an immediate impact on the cost of the same hospital stay. In this real-world study, we examined the hospitalization cost before and after the implementation of a goals-of-care program. We found that the overall cost in unselected medical patients did not differ significantly before and after program implementation; however, the cost of hospitalization decreased significantly among ICU patients by 17% after program implementation. This study highlights the potential value-based benefit of goals-of-care programs for patients who have serious illnesses.

**Abstract:**

The impact of goals-of-care programs on acute hospitalization costs is unclear. We compared the hospitalization cost in an 8-month period before implementation of a multimodal interdisciplinary goals-of-care program (1 May 2019 to 31 December 2019) to an 8-month period after program implementation (1 May 2020 to 31 December 2020). Propensity score weighting was used to adjust for differences in potential covariates. The primary outcome was total direct cost during the hospital stay for each index hospitalization. This analysis included 6977 patients in 2019 and 5964 patients in 2020. The total direct cost decreased by 3% in 2020 but was not statistically significant (ratio 0.97, 95% CI 0.92, 1.03). Under individual categories, there was a significant decrease in medical oncology (ratio 0.58, 95% CI 0.50, 0.68) and pharmacy costs (ratio 0.86, 95% CI 0.79, 0.96), and an increase in room and board (ratio 1.06, 95% CI 1.01, 1.10). In subgroup analysis, ICU patients had a significant reduction in total direct cost after program implementation (ratio 0.83, 95% CI 0.72, 0.94). After accounting for the length of ICU admission, we found that the total direct cost per hospital day was no longer different between 2019 and 2020 (ratio 0.986, 95% CI 0.92, 1.05), suggesting that shorter ICU admissions likely explained much of the observed cost savings. This study provides real-world data on how “in-the-moment” GOC conversations may contribute to reduced hospitalization costs among ICU patients.

## 1. Introduction

As cancer patients navigate the cancer journey, they are faced with a myriad of complex decisions and uncertainties. Goals-of-care (GOC) discussions aim to improve illness understanding and facilitate decision making while respecting patients’ expressed values and goals. GOC interventions have been found to be associated with improved outcomes, such as decreased intensive care unit utilization, 30-day unplanned readmission, and chemotherapy use at the end of life, and increased advance care plan documentation and hospice use [1,2,3,4,5,6].

Despite these benefits, the impact of GOC programs on the cost of care is unclear. On the one hand, these interventions are expected to reduce intensive measures that are not only inappropriate for some patients in the last months of life, but also costly for the healthcare system. On the other hand, the implementation of these programs may involve more clinical resources and many other factors may impact the cost of care beyond GOC discussions. The few studies that examined the financial impact of GOC discussions have all examined downstream acute care costs and reported some improvement with GOC interventions [7,8,9].

To our knowledge, no study has specifically examined the impact of a GOC program on hospitalization cost within the same admission. We hypothesize that effective serious illness conversations would reduce the overall hospitalization cost by decreasing acute care utilization, such as reducing the duration of intensive care unit admissions for selected patients. A better understanding of the cost implications of GOC programs may help hospital administrators assess the value of these programs and their sustainability. In response to the COVID-19 pandemic, the University of Texas MD Anderson Cancer Center implemented a multicomponent interdisciplinary GOC (myGOC) program in March 2020 with the goal of reducing intensive care unit utilization and improving the quality of care [3,10]. We found that the implementation of the myGOC program was associated with a reduced ICU mortality rate of 6.3% and shortened average ICU length of stay by 1.4 days [3]. In this propensity score weighted analysis of real-world data, we examined the impact of the myGOC program on hospitalization cost.

## 2. Methods

### 2.1. Study Design

This is a pre-planned secondary analysis of a study to examine the impact of our myGOC program on hospital and patient outcomes. Details of the study design and myGOC program have been described previously [3,4,10,11,12,13]. Because of the nature of this complex system-level intervention, randomized designs are not possible and thus we adopted a quasi-experimental design which allowed us to examine real-world data while accounting for co-variates with propensity score weighing. We compared the outcomes in an 8-month period before implementation of the myGOC program (1 May 2019 to 31 December 2019) to an 8-month period after implementation (1 May 2019 to 31 December 2019). Propensity score weighing was used to adjust for differences in potential covariates. This retrospective study protocol was approved by the Institutional Review Board at The University of Texas MD Anderson Cancer Center, with a waiver of informed consent.

### 2.2. Eligibility Criteria

This study included consecutive adult (age 18 years or greater) medical patients who were admitted to The University of Texas MD Anderson Cancer Center in the two pre-defined 8-month periods. A small proportion of the patients had repeated admissions during each cohort period (*n* = 2802 in 2019, *n* = 2264 in 2020). For these individuals, we randomly selected a single hospitalization to keep the observations independent.

### 2.3. myGOC Program

Details the of myGOC program have been published elsewhere [3,10,11,13]. Briefly, the myGOC program is an institute-wide initiative involving hospitalist service, palliative care, medical oncology, hematology, surgical oncology, emergency medicine, intensive care, nursing, case management, social work, chaplaincy, clinical ethics and senior leadership. The myGOC program has 6 key components: (1) identification of patients with advanced cancer who were at a high risk of mortality based on prognostic factors; (2) alerting the oncology team to have GOC conversations with these high-risk individuals during the same admission. Such conversations typically include prognostic disclosures, exploration of patients’ values, fears and goals, and discussion of healthcare decisions such as chemotherapy use, intensive care admission, resuscitation status, hospice referral and care planning [14,15,16,17,18,19]. The nature, frequency and intensity of these conversations varied widely by patients and oncologists; (3) the monitoring of GOC conversations in real time with daily reports; (4) institution-wide education on GOC conversations by the specialist palliative care team and real-time support provided by the GOC rapid response team for complex situations; (5) institutional committee monitoring GOC documentations and hospital outcomes longitudinally; and (6) strong institutional leadership endorsement of the myGOC program.

### 2.4. Data Collection

We retrieved baseline patient characteristics at the time of admission from electronic health records, including age, sex, race, ethnicity, cancer diagnosis, primary admitting service, admission type, COVID-19 infection status and SOFA score.

The primary outcome in this study was the total direct cost during the hospital stay for each index hospitalization. Direct costs were actual hospital expenses incurred as a result of patient care and were classified under the following categories: diagnostic imaging, emergency center, internal medicine, interventional radiology, laboratory/pathology, medical oncology, operations/anesthesia, pharmacy, professional charges, radiation oncology, room and board/supplies, surgical oncology and other. Cost data were retrieved by the Cost Management and Decision Support team using a top–down costing approach.

### 2.5. Statistical Analysis

The sample size was previously calculated for our primary outcome of ICU mortality [3]. With an estimated 600 medical ICU patients over both time periods and a baseline ICU mortality rate of 28%, we had 80% power to detect a 5% reduction in mortality using a two-tailed test at the 5% significance level.

We summarized the baseline patient characteristics with proportions, 95% confidence intervals (CIs), means, standard deviations, medians, and interquartile ranges (IQRs). Direct costs were expressed in January 2020 US dollars based upon the Consumer Price Index from the Bureau of Labor Statistics, Medical Care Services in US (city average, all urban consumers, not seasonally adjusted; https://beta.bls.gov/dataViewer/view/timeseries/CUUR0000SAM2 (accessed on 15 November 2023)).

Because of the proprietary nature of hospital charges, the primary statistic for the outcome was the model-adjusted direct cost ratio, defined as the direct costs in 2020 divided by direct costs in 2019. A cost ratio > 1 indicates higher cost in 2020 relative to 2019, and a ratio < 1 indicates lower cost.

This real-world data analysis included all hospitalized medical patients, regardless of their disease status or whether they had the GOC discussion. Because the two cohort populations overlapped by <1%, we treated them as independent cohorts. We applied propensity weighing to account for the differences in patient demographics between the two periods, which has been reported elsewhere [3]. Specifically, a logistic regression model was created with the outcome of interest being the probability of being assigned to the myGOC intervention and the independent variables being patient demographics (age, sex, race, and ethnicity), cancer diagnosis (solid tumor, hematologic malignancy, other, or unknown), SOFA score on admission and ICU admission during the index hospitalization (yes or no). The inverse proportion weights (IPW) were computed based on average treatment effect of the entire sample and used for adjustments in subsequent analyses. We examined the adequacy of propensity scores by assessing the percent reduction in standardized differences and the standardized difference between cohorts. We excluded 6 (0.2%) patients because of an inability to compute their propensity scores due to missing data and 125 (3.2%) patients because their propensity scores were extreme outliers. We examined the balance of the 2 cohorts by comparison of their histograms, and more formally with the standardized difference between cohorts. After applying IPWs, all standardized differences were <0.1 in magnitude. We used the same set of propensity scores for all cohorts examined for the study.

The weighted median and quartiles are reported for each study year. The direct cost was modeled with relation to the study year using a weighted generalized linear model with a gamma distribution and log link. The model-adjusted ratio between the years is reported with a 95% confidence interval. We conducted a subgroup analysis by cancer diagnosis given to patients with hematologic malignancies who often have a different clinical course and outcomes relative to those with solid tumors [20,21,22]. Because GOC discussions are most relevant for patients who are seriously ill, we also conducted a subgroup analysis by the ICU admission status.

The Statistical Analysis System (SAS version 9.4, SAS Institute, Cary, NC, USA) and R statistical software (R version 4.2.2, The R Foundation for Statistical Computing) were used for statistical analysis. Summary statistics and hypothesis testing were adjusted by inverse-weighted propensity scores utilizing the weight option in the SAS procedures and R survey package (version 4.1-1). A *p*-value of 0.05 or less was considered to be statistically significant for the primary outcome.

## 3. Results

### 3.1. Patient Characteristics

Patient characteristics have been reported previously [3]. This study included 6977 patients in 2019 and 5964 patients in 2020. After propensity weight adjustments, the key demographics were comparable before and after the intervention. Specifically, the mean age (SD) was 60.6 (14.6) in 2019 and 60.6 (14.8) in 2020; 44.4% in 2019 and 44.4% in 2020 were female; 62.7% in 2019 and 62.7% in 2020 were White, and 38.6% in 2019 and 38.5% in 2020 had a diagnosis of hematologic malignancy.

### 3.2. Direct Cost Ratios for All Hospital Admissions

Table 1 shows the adjusted mean hospitalization cost before and after myGOC program implementation. The total direct cost decreased by 3% and was not statistically significant (ratio 0.97, 95% CI 0.92, 1.03). Under individual categories, there was a significant decrease in medical oncology (ratio 0.58, 95% CI 0.50, 0.68) and pharmacy costs (ratio 0.86, 95% CI 0.79, 0.96), and an increase in room and board (ratio 1.06, 95% CI 1.01, 1.10). Under the pharmacy category, both costs related to systemic cancer therapies (ratio 0.82, 95% CI 0.67, 1.01) and non-systemic cancer therapies (ratio 0.96, 95% CI 0.90, 1.03) had a decrease, albeit non-statistically significant.

### 3.3. Direct Cost Ratios by Cancer Diagnosis

As shown in Table 2, the total direct cost did not differ significantly before and after myGOC program implementation for patients with hematologic malignancies (ratio 0.95, 95% CI 0.87, 1.03) and those with solid tumors (ratio 1.01, 95% CI 0.95, 1.08).

### 3.4. Direct Cost Ratios by ICU Admission Status

Of the 6977 (10.4%) hospitalized patients 727 in 2019 and 638 of 5964 (10.7%) patients in 2020 had an ICU admission. As shown in Table 3, among the patients who had an ICU admission, we observed a significant reduction in total direct cost after program implementation (ratio 0.83, 95% CI 0.72, 0.94). The cost of pharmacy, professional charges and room and board/supplies decreased significantly. In contrast, no significant change was found in the total direct cost between 2019 and 2020 in patients without an ICU admission (ratio 1.03, 95% CI 0.98, 1.09).

## 4. Discussion

In this propensity score analysis, we found that the overall hospitalization cost did not differ significantly after implementation of the GOC program. We observed a significant reduction in pharmacy cost, but this was balanced by a significant increase in room and board cost. In subgroup analysis, the overall hospitalization cost significantly reduced among patients who required an ICU stay. Taken together, our findings provide some insights regarding the potential impact of our myGOC program on hospitalization costs in the short term and its role in promoting value-based care.

Although multiple studies have reported on cost-savings with specialist palliative care referrals [23,24,25,26,27], only a handful have specifically examined the financial outcomes associated with GOC interventions, which may involve many other parties beyond palliative care. Zhang et al. conducted a secondary analysis of the Coping with Cancer Study and found that the mean cost of care was 36% lower in the last week of life among patients who reported having end-of-life discussions [7]. Patel et al. conducted a randomized trial to compare GOC discussions led by lay health worker and usual care. Patients on the intervention group had significantly lower healthcare costs in the last 30 days of life (USD 1048 vs. USD 23,482, *p* < 0.001), along with greater hospice use, fewer emergency department visits and few hospitalizations [8]. In a propensity score analysis of 35,154 patients, Starr et al. reported that patients who had a palliative care consultation to discuss GOC had lower acute care costs after discharge from index hospitalization compared to those who did not have a palliative care consultation for goals-of-care discussion, particularly among White patients [9]. In a quality improvement project, Lakin et al. assessed the impact of serious illness communication in primary care in 124 patients and reported that patients who had these conversations had significantly lower average monthly expenses over the last 6 months (USD 6297 vs. USD 8876, *p* = 0.04) and 3 months (USD 7263 vs. USD 11,406, *p* = 0.02) of life [28]. Taken together, the literature supports that GOC conversations can reduce the cost of end-of-life care by supporting proper care planning.

To our knowledge, our study is the first to examine a more immediate financial impact of GOC “in-the-moment” interventions. We previously found that patients who had an advanced care plan document during the index hospitalization had a significantly lower ICU mortality in 2020 compared to 2019 for both patients with hematologic malignancies (odds ratio 28% vs. 56%, *p* = 0.002) and solid tumors (23% vs. 49%, *p* < 0.0001), highlighting the importance of in-the-moment decision making in the context of the myGOC program [11]. Here, we found that the overall cost of care for all medical patients decreased only slightly and was not statistically significant. There could be multiple potential explanations. First, GOC interventions are particularly appropriate for patients with advanced cancer with a limited prognosis [29]. By examining costs for all admissions, we may be diluting the intervention effect. Second, GOC interventions represent a process and the intervention effect likely only applies after a direction on goals of care has been established (e.g., focus on comfort care under palliative care instead of life-sustaining measures in the ICU). By examining the entire hospital cost, the impact of GOC intervention may be dampened. Third, the intervention phase of this study occurred during the pandemic; despite propensity score weighting and adjustments for inflation, our analyses may not have fully controlled for all other factors that may impact total cost, such as rising nursing salaries.

Within the cost categories, we found that the medical oncology and pharmacy direct costs reduced significantly before and after implementation. This may potentially be related to a reduction in systemic therapy use and needs to be further confirmed [30]. Of note, the cost of cancer drugs has been rising over time, so this reduction is of particular interest. We also found an increase in room and board costs between the two time periods even after adjusting for inflation. This increase may be related to rising salaries during the pandemic.

Among patients who were admitted to the ICU, we found that hospitalization cost reduced significantly. This was consistent with our hypothesis that patients who were seriously ill were more likely to be impacted by the GOC intervention. We previously reported that GOC intervention was associated with a significant reduction of ICU length of admission from 6.8 days to 5.4 days (difference −1.4 days [−2.0, −0.7]; *p* < 0.001) [3]. After accounting for the length of ICU admission, we found that the total direct cost per hospital day was no longer different between 2019 and 2020, suggesting that shorter ICU admissions likely explained much of the observed cost savings. Of note, the pharmacy cost ratio remained significantly lower between 2019 and 2020, despite adjusting for the length of ICU admission.

This study has several limitations. First, this study was conducted in a single tertiary care cancer center and the findings may not be generalizable to other settings. Second, we only examined data in 8 months during 2019 and 8 months during 2020 to be consistent with our prior curated dataset. Third, although we used propensity score weighting to account for the potential differences in the baseline characteristics between the two study groups, there are likely confounders that we did not account for. Fourth, we were not able to restrict our main analysis to patients with advanced cancer and at a high risk of mortality because it was not possible to identify such patients in the 2019 cohort. We previously reported that 2918 (48.9%) of the patients in the 2020 cohort had advanced care plan documentation during the index hospitalization [3]. These patients likely had GOC discussions to a varying extent. The ICU subgroup analysis provided some insights into the program’s potential impact. Fifth, we only examined the cost ratio instead of actual costs. Sixth, because the GOC discussion was a process rather than a single event, we were not able to determine how cost changed immediately before or after GOC discussions during the hospital visit. The costs were also based on the whole admission, when the GOC intervention is likely to have the biggest impact on the acute care utilization after patients have made a decision. Seventh, we were unable to capture the actual cost of the myGOC program because this complex institutional intervention involved a large number of individuals in many departments. Detailed prospective mapping is required to properly account for the costs associated with program coordination and time for GOC discussions. Eighth, this study focused on costs only. We previously reported that ICU utilization decreased with myGOC implementation, which may explain part of the cost reduction [3]. A better understanding of myGOC’s impact on other services (such as chemotherapy use) would be helpful.

## 5. Conclusions

This study adds to the body of literature by providing real-world data on how “in-the-moment” GOC conversations, when delivered in a system-wide manner, may have a positive impact on the cost of care for cancer patients who required an ICU admission, ultimately promoting improved patient-centered and value-based care at the end of life.

## Figures and Tables

**Table 1:** Hospitalization direct cost ratios before (2019) and after (2020) the implementation of the goals-of-care program *.

Direct Cost	Cost Ratio (95% CI)	*p*-Value
Total	0.97 (0.92, 1.03)	0.33
Categories		
Diagnostic imaging	1.11 (0.94, 1.31)	0.23
Emergency center	1.00 (0.99, 1.01)	0.99
Internal medicine	0.99 (0.95, 1.02)	0.47
Interventional radiology	1.02 (0.99, 1.06)	0.23
Laboratory/pathology	1.06 (1.01, 1.11)	0.027
Medical oncology	0.82 (0.77, 0.88)	<0.0001
Operations/anesthesia	1.07 (0.98, 1.16)	0.12
Pharmacy	0.87 (0.79, 0.97)	0.008
Cancer therapy	0.82 (0.67, 1.01)	
Non-cancer therapy	0.96 (0.90, 1.03)	
Professional charges	0.997 (0.96, 1.04)	0.89
Room and board/supplies	1.05 (1.01, 1.10)	0.03
Surgical oncology	1.00 (0.998, 1.00)	0.43
Other	0.98 (0.93, 1.03)	0.36

Abbreviations: CI, confidence interval. * A cost ratio <1 suggests lower cost in 2020 vs. 2019, and vice versa.

**Table 2:** Hospitalization direct cost ratios before (2019) and after (2020) the implementation of the goals-of-care program by cancer diagnosis *.

	Total Direct Cost Ratio (95% CI)	
Direct Cost	Hematologic Malignancies	Solid Tumors
Total	0.95 (0.87, 1.03)	1.01 (0.95, 1.08)
Categories		
Diagnostic imaging	0.996 (0.98, 1.01)	1.17 (0.91, 1.51)
Emergency center	0.997 (0.98, 1.02)	1.001 (0.99, 1.01)
Internal medicine	0.99 (0.95, 1.04)	0.98 (0.94, 1.03)
Interventional radiology	1.00 (0.998, 1.00)	1.04 (0.98, 1.10)
Laboratory/pathology	1.06 (0.987, 1.14)	1.06 (1.01, 1.11)
Medical oncology	0.67 (0.60, 0.75)	1.01 (0.98, 1.04)
Operations/anesthesia	1.02 (0.95, 1.10)	1.09 (0.97, 1.21)
Pharmacy	0.87 (0.76, 0.99)	0.90 (0.82, 0.99)
Professional charges	1.01 (0.95, 1.08)	0.99 (0.94, 1.04)
Room and board/supplies	1.05 (0.988, 1.13)	1.05 (0.99, 1.11)
Surgical oncology	0.999 (0.996, 1.00)	1.00 (1.00, 1.001)
Other	0.95 (0.88, 1.03)	0.995 (0.93, 1.06)

Abbreviations: CI, confidence interval. * A cost ratio <1 suggests lower cost in 2020 vs. 2019, and vice versa.

**Table 3:** Hospitalization direct cost ratios before (2019) and after (2020) the implementation of the goals-of-care program by intensive care unit admission status *.

	Total Direct Cost Ratio (95% CI)		Average Direct Cost Ratio (95% CI) Per Hospital Day	
Direct Cost	ICU Admission	No ICU Admission	ICU Admission	No ICU Admission
Total	0.83 (0.72, 0.94)	1.03 (0.98, 1.09)	0.99 (0.92, 1.05)	0.99 (0.97, 1.02)
Categories				
Diagnostic imaging	0.99 (0.96, 1.02)	1.12 (0.93, 1.35)	1.005 (0.999, 1.01)	1.04 (0.98, 1.10)
Emergency center	1.05 (1.01, 1.08)	0.99 (0.98, 1.01)	1.03 (0.99, 1.06)	0.99 (0.98, 0.997)
Internal medicine	0.92 (0.79, 1.08)	0.998 (0.97, 1.03)	0.996 (0.98, 1.01)	0.99 (0.98, 1.001)
Interventional radiology	1.09 (1.01, 1.18)	1.01 (0.97, 1.06)	1.07 (1.01, 1.13)	1.002 (0.99, 1.02)
Laboratory/pathology	0.88 (0.77, 0.999)	1.13 (1.08, 1.18)	1.01 (0.98, 1.04)	1.02 (1.01, 1.04)
Medical oncology	0.85 (0.72, 1.01)	0.82 (0.77, 0.88)	0.996 (0.98, 1.02)	0.99 (0.99, 0.99)
Operations/anesthesia	1.30 (0.98, 1.73)	1.03 (0.95, 1.12)	1.05 (1.02, 1.08)	1.01 (0.99, 1.02)
Pharmacy	0.72 (0.58, 0.91)	0.93 (0.84, 1.04)	0.88 (0.78, 0.99)	0.93 (0.89, 0.97)
Professional charges	0.85 (0.77, 0.95)	1.06 (1.02, 1.09)	0.97 (0.91, 1.04)	0.999 (0.99, 1.01)
Room and board/supplies	0.90 (0.80, 1.01)	1.11 (1.06, 1.15)	1.05 (1.01, 1.09)	1.04 (1.03, 1.04)
Surgical oncology	1.00 (0.997, 1.01)	0.999 (0.998, 1.00)	1.00 (1.00, 1.00)	1.00 (1.00, 1.00)
Other	0.86 (0.75, 0.99)	1.02 (0.98, 1.07)	1.002 (0.99, 1.02)	0.999 (0.996, 1.002)

Abbreviations: CI, confidence interval; ICU, intensive care unit. * A cost ratio <1 suggests lower cost in 2020 vs. 2019 and vice versa.

## Data Availability

Individual participant data that underlie the results reported in this article after deidentification are available immediately and ending 5 years following publication to investigators whose proposed use of the data has been approved by the institutional review broad at the MD Anderson Cancer Centre to achieve the aims in the approved proposal. Proposals should be directed to the corresponding author. To gain access, data requestors will need to sign a data access agreement and provide funding support for data retrieval.
